# Bias in estimates of alcohol use among older people: selection effects due to design, health, and cohort replacement

**DOI:** 10.1186/s12889-015-2114-6

**Published:** 2015-08-11

**Authors:** Susanne Kelfve, Kozma Ahacic

**Affiliations:** Aging Research Center, Karolinska Institutet & Stockholm University, Gävlegatan 16, Stockholm, SE-113 30 Sweden; Department of Sociology, Stockholm University, Stockholm, Sweden; Centre for Epidemiology and Community Medicine, Health Care Services, Stockholm County Council, Stockholm, Sweden; Institute of Gerontology, School of Health Sciences, Jönköping University, Jönköping, Sweden; Department of Public Health Sciences, Karolinska Institutet, Stockholm, Sweden

## Abstract

**Background:**

There is a growing awareness of the need to include the oldest age groups in the epidemiological monitoring of alcohol consumption. This poses a number of challenges and this study sets out to examine the possible selection effects due to survey design, health status, and cohort replacement on estimates of alcohol use among the oldest old.

**Methods:**

Analyses were based on three repeated cross-sectional interview surveys from 1992, 2002 and 2011, with relatively high response rates (86 %). The samples were nationally representative of the Swedish population aged 77+ (total *n* = 2022). Current alcohol use was assessed by the question “How often do you drink alcoholic beverages, such as wine, beer or spirits?” Alcohol use was examined in relation to survey design (response rate, use of proxy interviews and telephone interviews), health (institutional living, limitations with Activities of Daily Living and mobility problems) and birth cohort (in relation to age and period). Two outcomes were studied using binary and ordered logistic regression; use of alcohol and frequency of use among alcohol users.

**Results:**

Higher estimates of alcohol use, as well as more frequent use, were associated with lower response rates, not using proxy interviews and exclusion of institutionalized respondents. When adjusted for health, none of these factors related to the survey design were significant. Moreover, the increase in alcohol use during the period was fully explained by cohort replacement. This cohort effect was also at least partially confounded by survey design and health effects. Results were similar for both outcomes.

**Conclusions:**

Survey non-participation in old age is likely to be associated with poor health and low alcohol consumption. Failure to include institutionalized respondents or those who are difficult to recruit is likely to lead to an overestimation of alcohol consumption, whereas basing prevalence on older data, at least in Sweden, is likely to underestimate the alcohol use of the oldest old. Trends in alcohol consumption in old age are highly sensitive for cohort effects. When analysing age-period-cohort effects, it is important to be aware of these health and design issues as they may lead to incorrect conclusions.

## Background

Monitoring the alcohol consumption of the population is an important tool for policymakers and researchers. Although several countries have recently reported increasing alcohol consumption among older persons [1–3], this segment of the population is often neglected in ongoing public health surveys [4]. In this article we consider some of the methodological challenges involved in monitoring the alcohol consumption of older people and the possible bias that can occur.

In Sweden [5] and sometimes elsewhere [6–9] trends in alcohol consumption are often monitored by surveys with response rates of around 50 % or below. Most studies suggest that alcohol consumption in the non-response group is larger than that in the group of people who respond [10–13]. However, the case may be different among elderly people.

A key issue in surveys of older people’s alcohol consumption is the health of the target group – as health is related to the probability of them being included in the sample [14, 15], their ability to participate in the survey [15] and their alcohol consumption [16, 17]. One of the main challenges is to obtain a representative sample that accurately reflects the entire population, without selection or non-response that favours a particular section of the population.

First, a relatively high proportion of the oldest old live in institutions. For a variety of reasons it is common to exclude those living in institutions from general population surveys [14]. While excluding this group may be a negligible problem in younger populations, there is a substantial part of the elderly population with a higher level of health problems that necessitate them being cared for in an institutional setting [15]. Hence, a survey that aims to assess alcohol consumption in the total population, will omit a substantial part of the oldest old population if institutionalized persons are excluded. Second, in the oldest age group some sampled persons will be unable to participate in the survey because of poor health or cognitive impairment. Extensive fieldwork is therefore needed to achieve a representative study population. This may require a variety of data collection methods, such as mixed interview modes. For example, whereas resistance to having a stranger in one’s home may be overcome by a telephone interview, hearing problems may be less problematic in a face-to-face interview than in a telephone interview, [18–20]. In addition, when gathering information about individuals who are difficult to recruit because of poor health, it is necessary to rely on proxy respondents, such as close relatives, who can answer on behalf of the person [15, 20, 21].

Another important source of error when estimating levels of alcohol consumption, or period trends in these levels, is the presence of cohort effects. Cohort effects refer to historical changes that occurred a shorter or longer time before the study took place, and which lead to differences between birth cohorts. Over time, each birth cohort at a specific age is replaced by later born cohorts, so-called cohort replacement. For example, whereas prevalence rates for ages 75+ in 2000 are for cohorts born 1925 or earlier, the same prevalence rates from 2010 are for cohorts born 1935 or earlier, that is, partially different cohorts. Since prevalence estimates are usually somewhat dated, the cohort replacement means that the birth cohorts within the age range of interest have also changed. Since older individuals have lived longer than younger individuals they have been exposed to a greater number of possible influences during their life time, i.e., they had a longer exposure time. Thus the likelihood of finding cohort effects should be greater in higher age groups. Depending on the source of the influences, such bias may take different forms and affect trend evaluations in a variety of ways. We have previously shown that the trend of decreasing abstention among elderly individuals in Sweden is entirely due to generational differences that probably have their origin in a rationing of alcohol in Sweden which ended in the mid-1950s [22]. Although cross-sectional analyses of the Swedish data indicated increasing abstinence rates with advanced age, longitudinal analyses indicated stable abstinence rates within cohorts. At the same time, this effect was only present among the generations born before the 1940s and therefore it did not affect the consumption trends in the younger population. Examples of trends in alcohol consumption being obscured by different sorts of cohort effects have also been found in several other countries, for example in the US [23] and Finland [24].

Furthermore, estimation of these cohort effects is likely to be intertwined with survey design and non-response issues. Our study of abstention used two samples; the methods to increase response rates described above (proxy interviews, mixed-mode etc.) were used for only one of the samples. Results suggested that analyses of the sample that did not use these methods underestimated the prevalence of abstinence and that cohort effects were also underestimated. The difference between these two survey approaches was not the focus of our earlier study and therefore we have decided to revisit the survey material concerning the oldest old. The present study aims to examine the effects of design, health and cohort differences, and their respective interrelationships, with estimates of alcohol consumption among the oldest old.

## Methods

### Material

The analysis was made possible through the use of a survey with high coverage of the target population, namely the Swedish Panel Study of Living Conditions of the Oldest Old (SWEOLD) [25]. This is a nationally representative survey targeting people aged 77 years and older. The design includes a mixed mode approach and proxy interviews, which allow the effect of these interview methods and the hypothetical effect of a low response rate to be modelled. Our material includes data from three cross-sectional waves from 1992, 2002 and 2011, with the non-response rates of 4.6, 15.6, and 13.8 % respectively. Item specific non-response also reduced the total study sample from 2061 to 2022 individuals, and the overall response rate was 86.1 %.

In SWEOLD a great deal of effort was put into obtaining a representative picture of the oldest old population and to including individuals regardless of their living situation, physical or cognitive status. A random sampling process was facilitated by using the Swedish system of personal identification numbers, which ensured a known sample inclusion probability for all individuals 77 years and older in Sweden.

Ethical approval was obtained by Uppsala University Hospital Ethical Committee, Dnr. 247/91 (SWEOLD 1992), Karolinska Institutet Ethical Research Committee, Dnr. 03–413 (SWEOLD 2002) and Regional Ethical Review Board in Stockholm, Dnr. 2010/403-31/4 (SWEOLD 2011).

### Independent variables

The independent variables used were grouped into three areas: variables related to survey design, health, and birth cohort. In each of these areas three variables were studied.

#### Survey design

Three aspects of survey design were modelled empirically: 1) whether the interview was conducted by telephone or face-to-face; 2) whether the sampled persons themselves answered or whether a proxy answered on their behalf; and 3) interview succession, i.e., the order in which sampled persons were interviewed during fieldwork. The first two variables were dichotomous, and the third was continuous.

The data collections started with an introductory letter being sent out to all individuals in the sample. This was followed up with a phone call during which an interviewer booked an appointment for the interview. Participants were primarily interviewed in person during a home visit, but other types of interviews were used when necessary. Proxy interviews were performed when the older person’s health status was too poor or the person was too physically or cognitively impaired to participate. Proxy interviews were conducted with a close relative, or in a few cases, with a professional caregiver. Proxy interviews were primarily conducted by telephone. Telephone interviews were also used when a respondent declined a home visit.

*Interview succession* refers to the relative position of each interview during the fieldwork, from those who were interviewed early in data collection to those who were never interviewed. With the assumption that those interviewed late (or never) were harder to recruit, this order corresponds to how response rates would have been if efforts to recruit had been more limited and the remainder of the sample (all of whom were subsequently interviewed) were non-responders. Interview succession was divided by ten to estimate a 10 % change in response rate. The measure provides a calculation of what an additional response would have added in terms of changing the estimate, e. g., having a response rate of 60 instead of 50 % etc. This approach is sometimes used to calculate the behaviour of non-responders. It then basically hypothesizes that sub-group analyses of the interviewed sample provide information about the alcohol consumption of the non-responders [10] along the line of a “continuum of resistance”, i.e., that harder to recruit individuals (those interviewed later in the fieldwork process) are more similar to the non-responders than easy to recruit individuals (those interviewed early in the fieldwork process) [26].

#### Health

Three dichotomous indicators of health were studied: physical mobility, activities of daily living (ADL), which refers to the ability to manage everyday tasks, and institutionalization. Having a *mobility problem* was defined as having at least one self-reported mobility problem (inability to walk 100 meters fairly briskly or inability to walk up and down stairs without difficulty). *ADL limitation* was defined as self-reported difficulty in carrying out one or more ADL tasks (eating, managing the toilet, dressing, getting up/going to bed or washing one’s hair). The third health variable was whether the person was living in an institution or in ordinary housing. *Living in an institution* refers to living in any type of nursing home with service around the clock. This variable has also been considered a design variable, i.e., indicating whether this group is included or not. In Sweden, people only move to nursing homes after a needs assessment by an assessor from the municipality. People with less severe care needs are cared for at home by home help services, which are also financed by taxes. Therefore, institutional care predominantly reflects an older person’s functional ability.

#### Birth cohorts

Two variables are interrelated with cohort: age and time period. *Period* was measured by the interview year (1992, 2002 and 2011). To make the birth year (cohort) and age-at-interview more comparable to the period estimates they were divided by ten, as there were approximately 10 years between the interview waves.

### Dependent variables

Two separate outcomes were studied: *Alcohol consumers* in contrast to abstainers and *Frequency of use among alcohol consumers.* The first was dichotomous and the second, excluding abstainers, could take three possible values: seldom (less than once a month, but at least once a year), monthly (less than once a week, but at least once a month), or weekly (at least once a week). The two dependent variables were created from the seven available options for answering the question “How often do you drink alcoholic beverages, such as wine, beer or spirits?” (5–7 days/week, 3–4 days/week, 1–2 days/week, 2–3 times/month, 1 time/month, 1–6 times/year, never).

### Analyses

First, all the bivariate relationships were modelled. Then the co-variation of the variables within each of the areas was evaluated: in model 1 we entered interview succession, proxy and telephone simultaneously to evaluate which of these survey design factors exhibited the strongest independent relationship with alcohol use. In model 2, we assessed whether institutionalization was associated with alcohol use independently of ADL limitation and mobility problem. Because we found a significant interaction effect between living in an institution and sex *(p = 0.001)*, two dichotomous variables for institutional living were included in the regression models: *living in an institution (men)* and *living in an institution (women)*. In model 3 we estimated the extent to which the cohort differences contributed to “explaining” the period changes, by entering period and cohort in the same models. Age was only presented in the bivariate analyses and was never entered in the models as a previous analysis of age, period, and cohort (apc) had shown that including all three variables simultaneously in one model was problematic [22]. Consequently, in that previous analysis we had to rely on a graphical solution. Owing to the lack of space and the small age span included, this approach could not be used here.

To evaluate the extent to which poor health explained the relationship of the survey design on alcohol use, the blocks of health and design variables were then entered together in one model (Model 4).

In the final model (Model 5), we tested if the effect of birth cohort on time trends in alcohol use were related with survey design and health.

Since women typically consume less alcohol than men [1], all models were adjusted for sex. To correct for different inclusion probabilities (there was an oversampling of the oldest age groups 2011 (85–99 for men and 90–99 for women), sample weights were used in all analyses, see Table 1.

**Table 1:** Descriptive statistics

	n	Weighted n	Weighted %
Period (response rate)			
1992 (95.4 %)	532	587.0	32.3
2002 (84.4 %)	610	610.0	33.5
2011 (86.2 %)	880	621.7	34.2
Sex			
Women	1173	1104	60.7
Men	849	714	39.3
Age			
Range	77–101	77–101	
Mean	84.6	83.0	
Birth year			
Range	1894–1934	1894–1934	
Mean	1918	1918	
Living in an institution			
Yes	312	226.8	12.5
No	1710	1591.9	87.5
ADL limitation			
Yes	619	495.5	27.3
No	1403	1323.2	72.8
Mobility problem			
Yes	1130	965.5	53.1
No	892	853.2	46.9
Proxy interview			
Yes	384	310.8	17.1
No	1638	1508.0	82.9
Telephone interview			
Yes	434	351.2	19.3
No	1588	1467.5	80.7
Alcohol consumption			
Abstainers	729	655.4	36.0
Seldom	485	441.4	24.3
Monthly	380	338.9	18.6
Weekly	428	383.1	21.1
Total	2022	1818.7	100

Binary logistic regressions were used for the dichotomous outcome alcohol use. For the ordinal outcome frequency of use among alcohol consumers, ordered logistic regression was used. The software used was Stata13 [27].

Finally, to illustrate the possible biasing effect on prevalence rates two figures are presented. The figures are based on the results from two logistic regressions models in which estimates were recalculated into prevalence numbers, to show rates instead of odds. The two studied outcomes were alcohol use and consuming alcohol on a weekly or more frequent basis in 1992, 2002 and in 2011. The first line of the overtime trend in each figure presents estimates from a model where the independent variables were interview succession and period change, with an adjustment for the lower prevalence among those living in institutions. Here the estimates were for a response rate corresponding to 50 %. The second line presents alternative estimates from the same model, although here the estimated values corresponded to a 100 % response rate instead of 50 %, and there was no adjustment for institutionalization. The estimates for the third line correspond to those from the second line, with an additional adjustment for the cohort differences.

## Results

### Factors associated with alcohol use and frequency of use

Lower proportions of alcohol users were found among those interviewed later in fieldwork (measured by interview succession), those interviewed by proxy, and those interviewed by telephone (as shown in the first column of Table 2). Individuals with poor health, as measured by institutional living, ADL limitations and mobility problems, were also less likely to consume alcohol. There were higher proportions of alcohol users in later survey waves, in lower age groups and in later birth cohorts. Finally, a greater proportion of men than women reported alcohol use.

**Table 2:** The proportion of alcohol consumers aged 77+ years and the frequency of alcohol consumption among consumers by sex, interview characteristics, health, period, birth cohort and age

	% Consumers	% among consumers (*n* = 1163)		
		Seldom	Monthly	Weekly
Sex				
Women	58.1	46.9	28.7	24.4
Men	73.1	26.9	29.7	43.4
Interview succession %^a^				
<25	67.9	34.3	28.7	37.0
25 - <50	64.0	36.9	31.2	31.8
50–75	62.1	38.4	31.1	30.5
>75	60.0	46.5	22.6	30.9
Proxy interview				
Yes	47.0	58.2	22.0	19.8
No	67.5	35.0	30.2	34.8
Telephone interview				
Yes	52.4	51.2	23.4	25.4
No	66.7	35.5	30.2	34.3
Living in an institution (women)				
Yes	45.2	72.9	18.5	8.6
Now	60.1	43.9	29.9	26.3
Living in an institution (men)				
Yes	40.1	42.5	28.2	29.3
No	77.0	25.9	29.8	44.3
ADL limitation				
Yes	46.9	52.3	26.6	21.1
No	70.4	34.4	29.8	35.9
Mobility problem				
Yes	54.9	45.5	26.1	28.4
No	74.2	31.6	31.7	36.7
Period				
1992	58.8	42.6	31.6	25.8
2002	63.6	39.7	31.4	28.9
2011	69.2	32.6	25.1	42.3
Birth cohorts^b^				
1894–1914	58.45	39.66	35.02	25.32
1915–1924	62.39	43.64	24.87	31.49
1925–1934	73.23	29.78	27.78	42.44
Age groups^b^				
77–79	71.9	34.5	31.3	34.2
80–84	66.1	35.8	30.2	34.1
85+	54.8	44.8	25.3	29.9
Total	64.0	37.9	29.1	32.9
Weighted n	1819	441	339	383

^a^For the descriptive statistics, interview succession has been categorized as <25, 25 to <50, 50 to 75, >75. It was otherwise a continuous variable

^b^Birth cohort and age are given linear representation in the multivariate analyses, but are presented here categorical for descriptive reasons (three classes)

The frequency of alcohol use among consumers, as shown in the remaining columns of Table 2, followed a similar pattern; that is, factors associated with a lower prevalence of alcohol use were also associated with less frequent use.

### Relative importance of the factors

#### Alcohol consumer

The odds ratios in the first column of Table 3 (adjusted for sex) confirm the association of all the factors analysed with the use of alcohol. The odds for alcohol use were lower for those interviewed later in the fieldwork, those interviewed by telephone and those interviewed by proxy. All health indicators were also significantly associated with lower odds for alcohol use. In contrast, a higher prevalence of alcohol use was associated with later survey wave, later cohort and lower age.

**Table 3:** Sex-adjusted odds ratios of being an alcohol consumer rather than a non-consumer depending on survey design, health, and birth cohort

		Bivariate		Within categories		Model 4		Model 5	
		OR	p-value	OR	p-value	OR	p-value	OR	p-value
*Survey design*				Model 1					
Interview succession *(linear)*		**0.95**	0.011	0.97	0.142	0.97	0.169	0.98	0.330
Proxy		**0.45**	<0.001	**0.49**	<0.001	0.94	0.746	0.99	0.977
Telephone		**0.59**	<0.001	0.89	0.493	0.82	0.209	0.73	0.054
*Health*				Model 2					
Living in an institution (Women)		**0.55**	0.001	0.98	0.906	1.06	0.801	1.11	0.624
Living in an institution (Men)		**0.20**	<0.001	**0.39**	<0.001	**0.42**	0.002	**0.43**	0.002
ADL limitation		**0.40**	<0.001	**0.58**	<0.001	**0.60**	<0.001	**0.65**	0.005
Mobility problem		**0.45**	<0.001	**0.60**	<0.001	**0.61**	<0.001	**0.59**	<0.001
*Birth cohort*				Model 3					
Period	1992	1.00	ref	1.00	ref			1.00	ref
	2002	1.21	0.152	0.72	0.080			1.06	0.788
	2011	**1.61**	<0.001	**0.59**	0.039			1.07	0.795
Birth cohort *(linear)*		**1.44**	<0.001	**1.78**	<0.001			**1.31**	0.026
Age *(linear)*		**0.58**	<0.001	^a^					

Significant estimates (p<0.05) are in bold ^a^As age, cohort and period cannot be analysed in the same model, and because the period change was not related to changed age distribution over the years, age was excluded in the full model

In the three first models the variables within each category are modelled together in relation to alcohol use. To save space, these three first models are placed in the same column, although they are modelled separate. Model 1 indicates that the greater part of the association between alcohol use and later interview succession and telephone interview was related to proxy interviews. That is, proxy interviews were more commonly performed later in the field work or by telephone. However, after adjustment for health (Model 4), none of the design variables were significantly associated with alcohol use. Thus, the association between alcohol use and survey design can be explained by those respondents who were harder to recruit having some disability and being more likely to be living in an institution.

Model 2 show that, in women, the lower odds for alcohol use among those living in an institution were mainly explained by their higher odds for ADL limitations and mobility problems; the association between institutional living and alcohol use disappeared after adjustment for these factors. In contrast, among men, institutional living was an independent explanatory indicator of abstention from alcohol, regardless of health. Both ADL limitations and mobility problems were independently negatively associated with alcohol consumption, confirming that alcohol use was less common among older people with poor health, as measured by physical function.

Model 3 shows that cohort replacement explained the increase over time in the proportion of alcohol users during the study period. That is, new cohorts, now reaching old age, have higher proportions of alcohol users than their predecessors. Additional analyses (not shown here) showed that the estimated period differences did not change a great deal when adjusted for age only.

Finally, modelling all factors together indicated that the cohort effect on the period increase was at least partially confounded by health and survey design, as both the birth-cohort and period estimates changed with these controls (Model 5).

#### Frequency among consumers

Similar patterns were found for the frequency measure (Table 4). Among the consumers, being interviewed by proxy or by telephone was associated with less frequent alcohol use (in analyses adjusted for sex). Modelling the factors together revealed that the association between telephone interviews and less frequent consumption was explained by the high level of proxy interviews among those interviewed by telephone (Model 1). As in the alcohol-consumer model, this association disappeared when we adjusted for health (Model 4). Thus, estimates of frequency of alcohol use are also sensitive to proxy interviews, i.e., individuals with poor health and less frequent consumption are likely to become non-responders if proxy interviews are not offered. Interview succession was not significantly associated with consumption frequency in the alcohol-consumer group. The results also indicated that for women, living in an institution is the strongest predictor of lower frequency of alcohol use, as it was significantly associated with less frequent alcohol use in all models.

**Table 4:** Sex-adjusted odds ratios for a more frequent^a^ alcohol consumption among alcohol consumers depending on survey design, health, and birth cohort

		Bivariate		Within categories		Model 4		Model 5	
		OR	p-value	OR	p-value	OR	p-value	OR	p-value
*Survey design*				Model 1					
Interview succession *(linear)*		0.96	0.079	0.98	0.353	0.97	0.289	0.99	0.585
Proxy		**0.44**	<0.001	**0.49**	0.001	0.77	0.293	0.80	0.359
Telephone		**0.62**	0.004	0.85	0.419	0.84	0.397	0.72	0.106
*Health*				Model 2					
Living in an institution (Women)		**0.29**	<0.001	**0.39**	0.002	**0.46**	0.019	**0.45**	0.020
Living in an institution (Men)		**0.49**	0.036	0.68	0.273	0.80	0.544	0.74	0.423
ADL limitation		**0.53**	0.000	0.77	0.127	0.81	0.219	0.86	0.400
Mobility problem		**0.66**	0.001	0.78	0.056	0.80	0.077	**0.77**	0.038
*Birth cohort*				Model 3					
Period	1992	1.00	ref	1.00	ref			1.00	ref
	2002	1.15	0.395	0.89	0.620			1.18	0.481
	2011	**1.88**	<0.001	1.16	0.625			**1.89**	0.044
Birth cohort *(linear)*		**1.40**	<0.001	**1.32**	0.036			1.04	0.801
Age *(linear)*		0.79	0.084	^b^					

Significant estimates (p<0.05) are in bold ^a^From ordered logistic regression models, which provide the average increase of the odds ratio for reporting one higher category, e.g. for weekly rather than monthly. The outcome had three levels (Seldom, monthly or weekly). The assumption of equal effect sizes (also called proportional odds/parallel lines) was tested with partial proportional odds models (Stata command gologit2). The assumption was not violated for any of the independent variables (*p* > =0.084 in model 5)

^b^As age, cohort and period cannot be analysed in the same model, and because the period change was not related to changed age distribution over the years, age was excluded in the full model

Cohort replacement also explained the period change between 1992 and 2011 for the frequency measure. In contrast to the consumer model, no cohort effect remained in the full model when controls for health and survey design were included (Model 5), thus indicating even stronger relationship with health and survey design for this outcome.

**Fig. 1:**
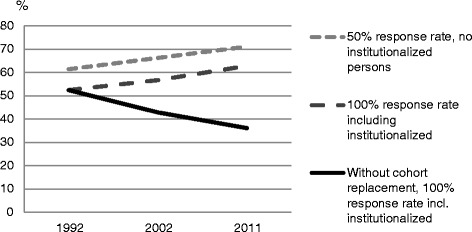
The prevalence of alcohol use in 1992, 2002 and 2011, with and without the estimated effect of non-response and cohort replacement

**Fig. 2:**
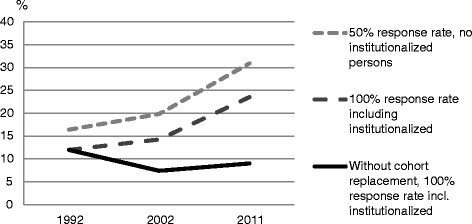
The prevalence of weekly alcohol use in 1992, 2002 and 2011, with and without the estimated effect of non-response and cohort replacement

Finally, Figs. 1 and 2 illustrates that, if the aim was to assess alcohol consumption in the total oldest old population, the proportion alcohol consumers, as well as the proportion weekly consumers, would have been be overestimated if the SWEOLD study had excluded people living in institutions and had subsequently had a response rate of 50 %. In addition, the trends over time would have been more or less stable, or negative, without the cohort replacement.

## Discussion

Estimates of old people’s alcohol use are higher among community-dwelling persons in good health. The efforts made in our data collection (proxy interviews, mixed-mode, etc.) led to higher response rates and lower estimates. This indicates that studies that focus on community-dwelling samples and do not use proxies may overestimate alcohol use in the elderly population. For example, Fornazar et al. [28] studied a community-dwelling, healthy population aged 80+ and found that 14–15 % were abstainers. Our study, that captured both institutionalized persons and many people outside institutions but in poor health, estimated that 36 % of the population aged 77+ were abstainers. The results showed that this underestimation applies to both alcohol use and the frequency of alcohol use. While increased survey efforts may lead to lower estimates, this lowering was fully explained by the poorer health of the people included in the survey as a result of these efforts. Lastly, the results also indicated that design and health issues may mislead analyses of age, period and cohort effects.

Moreover, the period changes between 1992 and 2011 were completely obscured by the cohort replacement. This is in line with previous analysis [22]. However, it was not only the proportion of the population using alcohol that increased due to the cohort replacement. The present results also suggest that this cohort effect obscures the trends over time in consumption levels, or at least, in the frequency of alcohol use. This result was in line with another apc-analysis of population’s alcohol consumption in Sweden [29]. Cohort effects in consumption levels are a topic that merits further investigation. Studies of average consumption levels in the US, and elsewhere, have indicated the presence of cohort effects [23, 24]. The effect due to this in period trends is however difficult to assess since age-period-cohort studies seldom present and compare the raw period scores with the adjusted ones. Cohort effects may lead to the incorrect attributing of trend changes in time, as historical influences on trends and trend changes can conceal the present development during the studied periods.

Our results differ from the results of studies on younger populations, which basically suggest that alcohol consumption levels are higher among non-responders than among responders [10–13]. In old age, our results indicate that the opposite is the case - that non-response is associated with abstention and less frequent alcohol use. One implication of this, i.e., that the association between alcohol consumption and non-response differs between age-groups, means that comparing older and younger age-groups even within the same study may be problematic and misleading.

In addition, our results indicate how easily survey design can skew age-period-cohort analyses. As a result of the potential collinearity, the solutions from age-period-cohort analyses are particularly sensitive to such effects.

The health of older people is highly relevant to survey participation. Our results showed that regardless of how poor health was measured – using institutional living, ADL limitations or mobility problems – it was associated with less alcohol use. The main association between alcohol use and survey design was driven by proxy interviews. This suggests that in a survey targeting the oldest old, it is above all the failure to use proxy interviews that will lead to an overestimation of the proportion of alcohol consumers and consumption frequency. Furthermore, previous research has suggested that exclusion of institutionalized individuals results in a negligible underestimation of alcohol consumption; as such individuals generally constitute a small fraction of the population [30]. In old age, this is not true however. In Sweden, for instance, a study restricted to community-dwelling individuals will inevitably exclude the 14 % of the 80+ population living in institutions [31] – a group that, according to our results, is characterized by significantly less alcohol use than the community-dwelling 80+ population.

According to our results, the association between institutional living and alcohol consumption differed by sex. Independent of their health status, women living in institutions consumed alcohol less frequently than the women living in ordinary housing, while fewer men living in institutions drank at all in comparison to other men. Men typically prefer to consume larger quantities than women, a drinking pattern that might be more difficult to sustain when circumstances change in old age. How institutionalization actually affects alcohol habits longitudinally is less clear, but it is an interesting question for further research.

Otherwise, the general direction of the relationship between health and alcohol use is probably such that healthy older persons are able to continue drinking as they have done previously rather than non-consumers being more likely to develop poor health. Moreover, selection effects probably contribute as people with serious alcohol problems are more likely to become abstainers over time [32].

### Strengths and limitations

The main strength of the present study is the high level of representativeness in the oldest part of the population—an interview group that normally demonstrates high levels of non-random non-response. However, SWEOLD is a multipurpose study, not primarily a study about alcohol, therefore, the alcohol questions that were included did not allow us to measure consumption volume.

The use of different sources and interview modes in SWEOLD can be considered not only a strength but also a limitation, as survey questions may be sensitive to the way an interview is conducted. The validity of proxy reports differs according to the type of question, but previous research supports the validity of proxy reports on an aggregated level in the assessment of alcohol consumption [33]. Greenfield, Midanik, & Rogers [34] have reported that telephone interviews provided an estimation of rates of self-reported alcohol consumption that were as satisfactory as those provided by face-to-face interviews, but no specific analyses for older age-groups has been done.

Exclusion of older people living in institutions may influence the results differently in different countries and over different time periods. The threshold for when an older individual moves into institutional care depends on several factors, such as the welfare system, current social policies, availability of institutional facilities, and the family situation [35, 36]. Additionally, institutional living may be associated differently with alcohol consumption depending on factors such as legislation and social attitudes (which affect the availability of alcohol for institutionalized or disabled individuals).

## Conclusions

Trends in alcohol consumption in old age are likely to be affected by cohort replacement. Basing prevalence rates on older data is likely to underestimate consumption, at least in Sweden. Moreover, a study of the oldest age groups that fails to include institutionalized individuals, those with poor health or those who are difficult to recruit, results in a higher proportion of consumers as well as higher estimates of drinking frequencies. Assuming that drinking status (consuming or abstaining from alcohol) and drinking frequency are closely connected with the amount of alcohol consumed, a likely consequence will be overestimated alcohol consumption in the oldest age groups.
